# A network perspective on the ecology of gut microbiota and progression of type 2 diabetes: Linkages to keystone taxa in a Mexican cohort

**DOI:** 10.3389/fendo.2023.1128767

**Published:** 2023-04-12

**Authors:** Diego A. Esquivel-Hernández, Yoscelina Estrella Martínez-López, Jean Paul Sánchez-Castañeda, Daniel Neri-Rosario, Cristian Padrón-Manrique, David Giron-Villalobos, Cristian Mendoza-Ortíz, Osbaldo Resendis-Antonio

**Affiliations:** ^1^ Human Systems Biology Laboratory, Instituto Nacional de Medicina Genómica (INMEGEN), Mexico City, Mexico; ^2^ Programa de Doctorado en Ciencias Médicas, Odontológicas y de la Salud, Universidad Nacional Autónoma de México (UNAM), Ciudad de México, Mexico; ^3^ Metabolic Research Laboratory, Department of Medicine and Nutrition, University of Guanajuato, León, Guanajuato, Mexico; ^4^ Programa de Maestría en Ciencias Bioquímicas, Universidad Nacional Autónoma de México (UNAM), Ciudad de México, Mexico; ^5^ Programa de Doctorado en Ciencias Biomédicas, Universidad Nacional Autónoma de México (UNAM), Ciudad de México, Mexico; ^6^ Coordinación de la Investigación Científica – Red de Apoyo a la Investigación, Universidad Nacional Autónoma de México (UNAM), Ciudad de México, Mexico; ^7^ Centro de Ciencias de la Complejidad, Universidad Nacional Autónoma de México (UNAM), Ciudad de México, Mexico

**Keywords:** gut microbiota, microbial ecology, systems biology, type 2 diabetes, network analysis

## Abstract

**Introduction:**

The human gut microbiota (GM) is a dynamic system which ecological interactions among the community members affect the host metabolism. Understanding the principles that rule the bidirectional communication between GM and its host, is one of the most valuable enterprise for uncovering how bacterial ecology influences the clinical variables in the host.

**Methods:**

Here, we used SparCC to infer association networks in 16S rRNA gene amplicon data from the GM of a cohort of Mexican patients with type 2 diabetes (T2D) in different stages: NG (normoglycemic), IFG (impaired fasting glucose), IGT (impaired glucose tolerance), IFG + IGT (impaired fasting glucose plus impaired glucose tolerance), T2D and T2D treated (T2D with a 5-year ongoing treatment).

**Results:**

By exploring the network topology from the different stages of T2D, we observed that, as the disease progress, the networks lose the association between bacteria. It suggests that the microbial community becomes highly sensitive to perturbations in individuals with T2D. With the purpose to identify those genera that guide this transition, we computationally found keystone taxa (driver nodes) and core genera for a Mexican T2D cohort. Altogether, we suggest a set of genera driving the progress of the T2D in a Mexican cohort, among them *Ruminococcaceae NK4A214* group, *Ruminococcaceae UCG-010, Ruminococcaceae UCG-002, Ruminococcaceae UCG-005, Alistipes, Anaerostipes*, and *Terrisporobacter*.

**Discussion:**

Based on a network approach, this study suggests a set of genera that can serve as a potential biomarker to distinguish the distinct degree of advances in T2D for a Mexican cohort of patients. Beyond limiting our conclusion to one population, we present a computational pipeline to link ecological networks and clinical stages in T2D, and desirable aim to advance in the field of precision medicine.

## 1 Introduction

Type 2 Diabetes Mellitus (T2D) is considered a global epidemic with a constant increase in new cases and negative economic and social impact on public health (1, 2). T2D is preceded by prediabetes (PreT2D), a condition characterized by intermediate hyperglycemia, insulin resistance (IR), and β-cell dysfunction that predisposes individuals to the development of T2D (3, 4). PreT2D can be prevented or delayed through lifestyle modifications, drugs, or bariatric surgery; however, up to 70% of people with preT2D will eventually evolve into T2D (4, 5). Current strategies for successfully diagnosing and managing preT2D are limited in part by an incomplete understanding of its pathophysiology (2).

Recently, human gut microbiota (GM) alterations were proposed as an important factor in the progression of T2D (6). Several cohorts and cross-sectional studies worldwide reported associations between the GM composition to preT2D and T2D (7). These studies indicated that several gut microorganisms (e.g., *Escherichia/Shigella, Ruminococcus, Dorea, and Veillonella*) (8) increase the absorption of energy from food, cause chronic low-grade inflammation, regulate fatty acid metabolism, secrete derived peptides, and increase the metabolic endotoxins production (lipopolysaccharides), which conduct into insulin resistance (IR). Furthermore, long-term IR leads to a constant raised level of systemic glucose concentration (9). According to these studies, a clear association between GM composition and T2D has been observed. However, bacterial genera associated with preTD2 and T2D differ when different populations are compared (8). For example, a Danish preT2D population suffers dysbiosis in the GM characterized by a decreased abundance of *Clostridium* genus and *Akkermansia muciniphila* strain (10). European and Chinese studies have demonstrated that the quantity of *Firmicutes*, *Bifidobacteria*, and *Clostridia* was significantly lower in T2D patients compared to healthy individuals, while the number of *Bacteroidetes* and *beta Proteobacteria* was markedly higher in both populations (11, 12). Likewise, in the Chinese cohort, the *Bacteroidetes/Firmicutes* ratio in T2D was positively and significantly correlated with plasma glucose concentration. Still, it appeared independent of body weight, confirming its association with reduced glucose tolerance (12). Moreover, a cross-sectional study from two Dutch population-based cohorts: the Rotterdam Study and the Lifelines DEEP study, reported associations among α diversity, β diversity, and taxa with the Homeostatic Model Assessment of Insulin Resistance (HOMA-IR) and with T2D. They reported 12 bacterial genera (butyrate producers) associated with HOMA-IR or T2D. (i.e., *Christensenellaceae, Christensenellaceae R7 group, Marvinbryantia, Ruminococcaceae UCG-005, Ruminococcaceae UCG-008, Ruminococcaceae UCG-010, Ruminococcaceae NK4A214 group, Clostridiaceae 1, Peptostreptococcaceae, Clostridium sensu stricto 1, Intestinibacter and Romboutsia*) (13).

The composition and structure of GM can be analyzed with specialized bioinformatics tools such as Sparse Correlations for Compositional data (SparCC), Sparse and Compositionally Robust Inference of Microbial Ecological Networks (SPEIC-EASI), and Bayesian Analysis of Compositional Covariance (BAnOCC) (14). Due to the high data complexity, these algorithms must be able to model the complex interactions and nonlinear effects between microbial communities (15). In a Mexican population with preT2D and T2D, Diener and collaborators studied 16S rRNA gene amplicon data from a cohort of 405 participants. They reported that *Escherichia* and *Veillonella* were associated with T2D progression, along with biochemical measures of blood glucose and insulin-related measures. Furthermore, *Blautia* and *Anaerostipes* were related to improved β-cell function and insulin efficiency, and these genera decreased with T2D development. Besides, the authors argue remarkable evidence that GM can alter intestinal inflammation (8).

A Chinese Cohort of 450 T2D subjects was exposed to two clinical interventions: metformin and AMC (Chinese herbal formula from *Rhizoma Anemarrhenae*, *Momordica charantia*, *Coptis Chinensis*, aloe vera, and red yeast rice). Authors reported GM alterations through SparCC association networks in the group treated with metformin. They observed a notable increase in *Blautia spp* (producer of short-chain fatty acids SCFA). Moreover, AMC treatment increased the abundance of two genera related to butyrate production (i.e., *Faecalibacterium* and *Roseburia*) (16, 17). All these results have contributed to a better understanding of the role of GM and T2D. However, these contributions remained at the association studies level without a deeper analysis of the ecological insights of gut microbial communities. Subsequently, medicine has emphasized reductionist ways, where the subunits of a system are analyzed separately, ignoring their complex non-linear interactions (18).

To understand the interactions and insights between GM and T2D patients, we studied the behavior of GM based on the concept of “the medical ecology of the human gut microbiome”. This concept relays on the need for new ecological perspectives and dynamical systems theory to advocate personalized medicine (19, 20). Furthermore, precision medicine has emerged with remarkable results in treating T2D Sommer and collaborators. (21) and collaborators reported differences in hemoglobin A1c (HbA1c) reductions between three T2D drugs (sitagliptin, pioglitazone, and canagliflozin) (21). According to their results, using simple clinical measures to identify the drug class most likely to deliver the greatest glycemic reduction for a given patient (22).

Bioinformatics, omics sciences, and systems biology have paved the way for the development of new strategies, to characterize a healthy and unhealthy microbiota composition and its relationship with the host (23). For example, Ezzamouri and collaborators used metagenomics data from a metformin study with genome-scale metabolic modelling of the key bacteria (e.g. *Akkermansia municiphila, Intestinibacter bartlettii, Clostridium saudiense, Romboutsia timonensis*) to research the mechanistic role of the GM in response to metformin in a Spanish T2D cohort (24). However, the GM is a complex ecological system that involves interactions between hundreds of bacterial species. Thus, scientific research should focus on studying complex networks of nonlinear interactions between many entities. Efforts to develop this field of medical ecology of the human GM have been reported (25, 26). Although specifically for Mexican T2D patients, our work represents the first attempt from a medical ecology perspective to understand the interactions between the GM and its host. For this reason, we used GM 16S rRNA gene amplicon data from a Mexican T2D cohort to perform systems biology and bioinformatics analysis between study groups (i.e., NG (Normoglycemic) IFG (Impaired Fasting Glucose), IGT (Impaired Glucose Tolerance), IFG+IGT (both conditions of preT2D), T2D (Type 2 diabetes), and T2D (Type 2 diabetes with 5 years ongoing treatment). Our specific objectives were to accomplish a: 1) GM ecological analysis (α-β diversity), 2) evaluation and analysis of association networks focused on their topological characteristics and their association with the clinical status, 3) differential abundance analysis at the genus level, and 4) supervised machine learning analysis to denoise GM data and improve the identification of keystone-taxa pattern shifts in Mexican T2D subjects.

Our results shed light on the role of several genera as driver nodes to explain the changes in the community structure from one stage to another, along with the development of preT2D and T2D within a Mexican cohort.

## 2 Materials and methods

### 2.1 Data collection and processing

**Figure 1:**
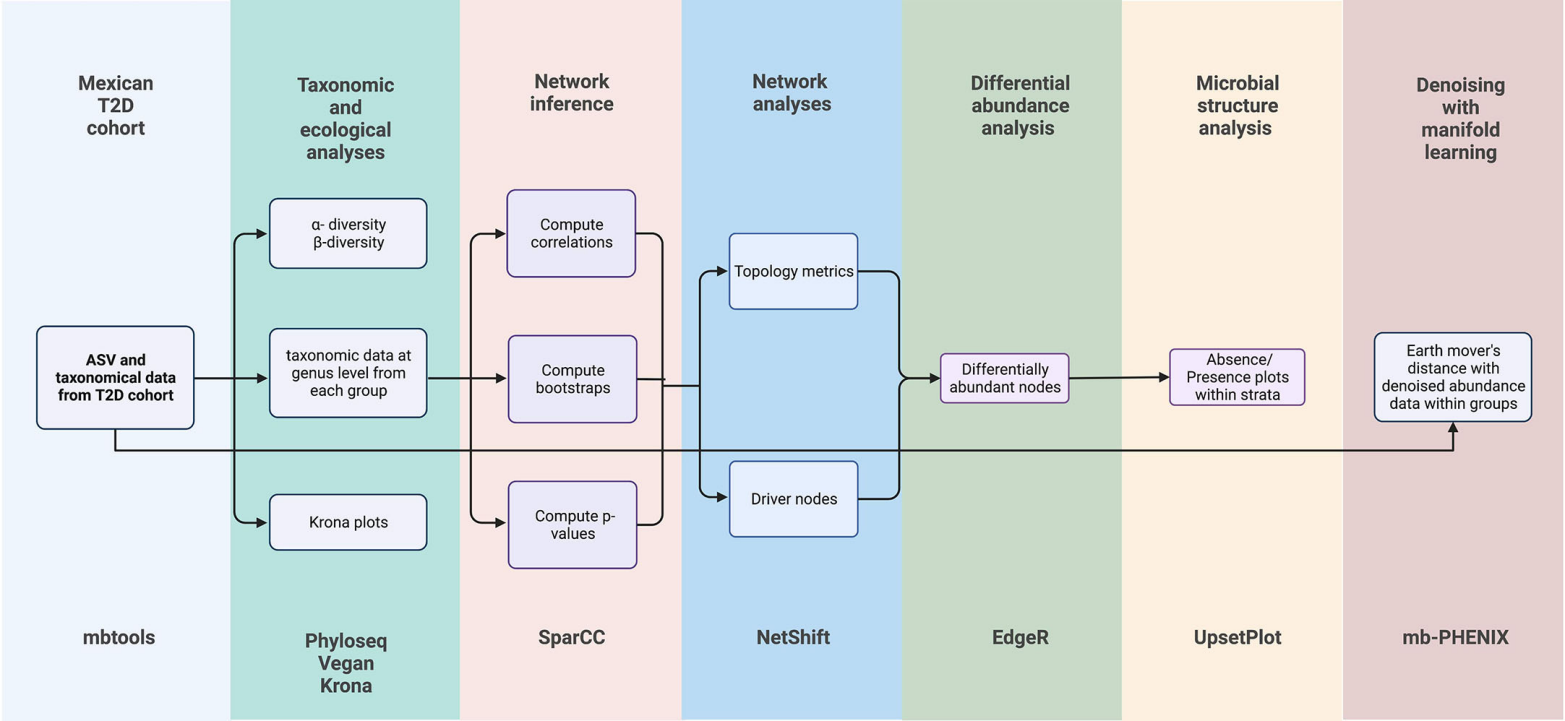
Experimental and Bioinformatics for taxonomic and ecological analyses, inference networks, network analyses, differential abundance analyses, microbial structure analyses, and supervised machine learning analysis. Bioinformatic tools are added at the bottom of the figure.

We performed an association network analysis with GM 16S rRNA gene amplicon data from a Mexican cohort of T2D patients (8, 27) (**Figure 1**). Specifically, we used variants.csv file (16S rRNA gene amplicon sequence variants ASV and their abundances in samples) and taxa.csv file (taxonomy assignment for each variant) from the GitHub repository https://github.com/resendislab/mext2d/tree/master/data. Then, we stratified all samples based on their T2D status as follows: 1) NG, 2) IFG, 3) IGT, 4) IFG+IGT, 5) T2D, and 6) T2D treated.

### 2.2 Taxonomic and ecological analysis

With the taxonomy assignment for amplicon sequence variants (ASVs), we prepared Krona plots (RRID: SCR_012785) to explore the microbial diversity of all samples (28). We constructed rarefaction curves using the R library vegan v2.4-6 (RRID: SCR_011950) (29) from R (version 4.0.4). A phyloseq object with ASV data was used to calculate α diversity indexes (i.e., Observed, Chao 1, Fisher, Simpson, Inv Simpson, and Shannon indexes), which were computed by R Phyloseq (RRID: SCR_013080) library 1.34.0 (30).

### 2.3 Differential abundance analysis

We used EdgeR (RRID: SCR_012802) (31) to assess differential abundance changes of the GM along different T2D stages. First, we filtered out OTUs for which the variance across all samples is very low (1e-5) and did this before ever passing the data to edgeR. Also, we estimated a series of log-linear generalized linear models predicting each ASV abundance. ASVs were considered differentially abundant at a false discovery rate (FDR) < 0.01. To examine potential keystone taxa at the genus level, we analyzed differentially abundant bacteria between two stages of T2D: NG vs IFG, IFG vs IGT, IFG+IGT vs T2D, and T2D vs T2D treated. Moreover, these pairwise comparisons were prepared to evaluate the changes in the GM composition in the subsequent stages, that is, step by step, until the frank diagnosis of T2D (27, 32). Finally, we performed an upset plot graph to describe intersections of differentially abundant genera between comparisons.

### 2.4 Network inference

We used the SparCC network inference approach (RRID: SCR_022734) (33) to infer underlying interactions from each group (i.e., NG, IFG, IGT, IFG+IGT, T2D, and T2D treated). Then, we utilized the non-normalized taxonomic abundance at the genus level to compute associations and prepared one network for each group. SparCC was run based on in-house scripts and adaptations from Netherlands Bioinformatics and Systems Biology Research School. First, we computed correlations (compositionality-robust) as the median of ten iterations, where SparCC averages its results over several estimates of the true fractions, with the Dirichlet distribution. Second, we calculated bootstraps and prepared one correlation matrix from one resampled dataset (n=100 iterations). Third, we computed p-values, based on bootstrapped correlation scores. Fourth, we selected nodes and edges based on the determined cut point of correlation level (0.60). The results from SparCC were plotted in an interactive network with VisNetwork R library (34) and were uploaded to Github (https://github.com/resendislab/MEXT2D_Networks).

### 2.5 Network analysis

To further evaluate the topological features of each network, we used Netshift software (RRID: SCR_022733) to identify driver nodes between case-control association networks (https://web.rniapps.net/netshift/) (35). We focused on identifying key attributes (e.g., nodes, clusters, and edges) based on a two conditions approach. NG vs IFG, IFG vs IGT, IFG+IGT vs T2D, T2D vs T2D treated. Then, we detected driver nodes from all comparisons.

### 2.6 Microbial structure analysis

Absence/presence plots were generated using the Upset R library (RRID: SCR_022731) (36) from the nodes network for each group (i.e., NG, IFG, IGT, IFG+IGT, T2D, and T2D treated).

### 2.7 Denoised microbiota data analysis by supervised machine learning

We denoised ASV data from the GM of a Mexican T2D cohort (https://github.com/resendislab/mext2d/tree/master/data) through a supervised denoising-machine learning approach named mb-PHENIX (37). We performed this analysis to observe the driver nodes involved in the transition of T2D states (37) and because of the high rate of missing information of microbiome data (38) that do not let find cluster structure with traditional unsupervised methods. The mb-PHENIX method consists of mapping different classes (here T2D stages) in the low dimensional space as far apart as possible, while maintaining the internal class structure and the inter-class relationships. Then, the missing taxa data is recovered (denoising) by sharing taxa information among the nearest neighbors.

We preprocessed the ASV table for imputation with the following steps: 1) We filtered the ASV by the number of counts above 20 which detection is deemed non-negligible and at least appears in 5 samples. 2) We performed L1 normalization. 3) Root squared transformation with the parameters of mb-PHENIX as follows: for PCA (n_components=100,random_state=1), then for the PCA space we applied supervised embedding with UMAP (n_components=2, verbose=True, metric=‘cosine’, n_epochs=1000, min_dist=0.1, random_state=1, n_neighbors=500, target_weight=0.5), and last the imputation *via* diffusion (t=5, decay=50, metric=‘euclidean’, knn=17). After imputation, we calculated the most significant taxa for each T2D stage within the imputed ASV data with the earth mover’s distance metric (EMD) (39). Particularly, we compared each T2D stage against the rest of the groups to quantify the differences between distributions among T2D stages. Then, the EMD was multiplied by the sign of the mean difference of each cluster to denote the overall direction of the shift (EMD score). Lastly, the imputed ASV table was collapsed to identify their taxonomical annotation at the genus level. We used EMD (40), a nonparametric measure of the distance between two distributions that quantifies the flow required to morph one distribution to another. It is defined as the L1 norm of the cumulative density functions, DEMD = \\CDF1 -CDF2\\1; and has successfully been used to quantify gene expression differences in single-cell data and microbiome data (39). Also, the EMD metric was used for imputed data *via* diffusion (41). Additionally, the EMD metric does not make parametric assumptions about their underlying distributions (41). The details of the mb-PHENIX software can be found in (https://github.com/resendislab/mb-PHENIX).

### 2.8 Linear discriminant analysis effect size

Linear discriminant analysis effect size (LEfSe) (RRID: SCR_014609), is a method that combines non-parametric Kruskal-Wallis and Wilcoxon rank sum test with linear discriminant analysis (LDA) (42). It was employed to detect the features in terms of bacterial genera to discriminate communities in each group (NG, IFG, IGT, IFG+IGT, T2D, and T2D treated). To run this analysis, we used the galaxy server from Hutlab (https://huttenhower.sph.harvard.edu/galaxy/) with the following parameters: 1) Alpha values for the factorial Kruskal-Wallis and pairwise Wilcoxon tests among classes were 0.05, 2) Threshold on the logarithmic LDA score for discriminative features was set to 2, and 3) Set the strategy for multi-class analysis was set to one against all.

## 3 Results

Classical approaches to analyzing microbiomes are based on taxonomic profiling and ecological diversity studies (e.g., phyloseq, edgeR, krona). Thus, we started with these analyses to describe the GM of a Mexican T2D cohort. To disentangle the complexity of the GM structure and ecological interactions on the progress of T2D in a Mexican cohort, we used a multidimensional approach based on bioinformatics tools such as SparCC, Netshift, and mb-PHENIX.

### 3.1 Profiling of 16S rRNA gene amplicon data and microbial diversity measurements of Mexican T2D cohort

Gut bacteria communities were highly diverse in all T2D samples, independently of their disease stage. The rarefaction curves (**Figure S1 A-F**) reach asymptotes in a range of 100-600 species (set size= 50) in all samples. We did not set up a threshold based on the lowest number of sequences found in a sample, because this would artificially mask the diversity of the community (43). As seen in the rarefaction curves, the NG patients had greater diversity across GM samples than the disease groups.

Related to GM taxonomy, the Krona graphs (https://github.com/resendislab/MEXT2D_Networks/tree/main/results) for NG patients showed that *Firmicutes* and *Bacteroidetes* ranged 40-80% and 20-60%, respectively. For IFG patients, *Firmicutes* ranged in 37-91% and 2-50% for *Bacteroidetes*. For IGT patients, *Firmicutes* ranged in 40-80% and 10-50% for *Bacteroidetes*. For IFG+IGT patients, Firmicutes ranged 30-70% and 10-35% for *Bacteroidetes.* For T2D patients, *Firmicutes* ranged from 40-60% and *Bacteroidetes* 5-30%. Lastly, T2D patients with treatment *Firmicutes* ranged 50-80% and *Bacteroidetes* 1-20%.

The Chao1, ACE, Shannon, and Fisher indexes (**Figure S2**) were calculated to estimate the α-diversity. No significant differences were found between the five groups, but IGT subjects showed a slightly increased diversity compared with all other stages. This result indicated that IGT patients had higher microbial diversity than in other stages. With this result, we can hypothesize that this diversity increase may be because of an internal feedback mechanism between the GM and its host as a last effort to stabilize the evolving microbial community due to the progression of T2D. This hypothesis is supported by the latest reports on the functional redundancy of microbial communities in the human gut and the holobiont theory as a framework of analysis for microbial communities associated with their host (44–46).

A previous study from our group reported that our Mexican T2D cohort had some degree of obesity (8, 27). For example, groups with IGT and T2D have the highest values of body mass index (BMI) compared with other groups such as NG and IFG (8). This behavior was also observed in other computational modeling studies (Flux Balance Analysis FBA) related to T2D cohorts (47).

To estimate β-diversity, we used non-phylogenetic methods such as Jaccard distances and Non-Metric Multidimensional Scaling (NMDS) plotting (48). The results showed undefined clustering patterns (**Figure S3A**). Moreover, we made an additional NMDS plot at phylum level to observe the differences among clustering groups. We observed the vast amplitude of members of *Firmicutes* phylum and reinforced the remarkable abundance of these members in the GM of humans (49) (**Figure S3B**). However, we did not observe clustering of the groups based on the T2D stage. Then, we established that more complex nonlinear (unsupervised dimensionality) reduction methods did not present separation patterns of samples based on T2D stages groups (**Figure S5A** UMAP). These results are likely due to technical noise, high-dimensionality, sparsity, and intrinsic ecological community heterogeneity (50). Therefore, it is necessary to use supervised methods to reveal the underlying topological information on these noisy and highly heterogeneous systems (38).

### 3.2 Differentially abundance analysis as a tool to discover keystone taxa among T2D stages

**Figure 2:**
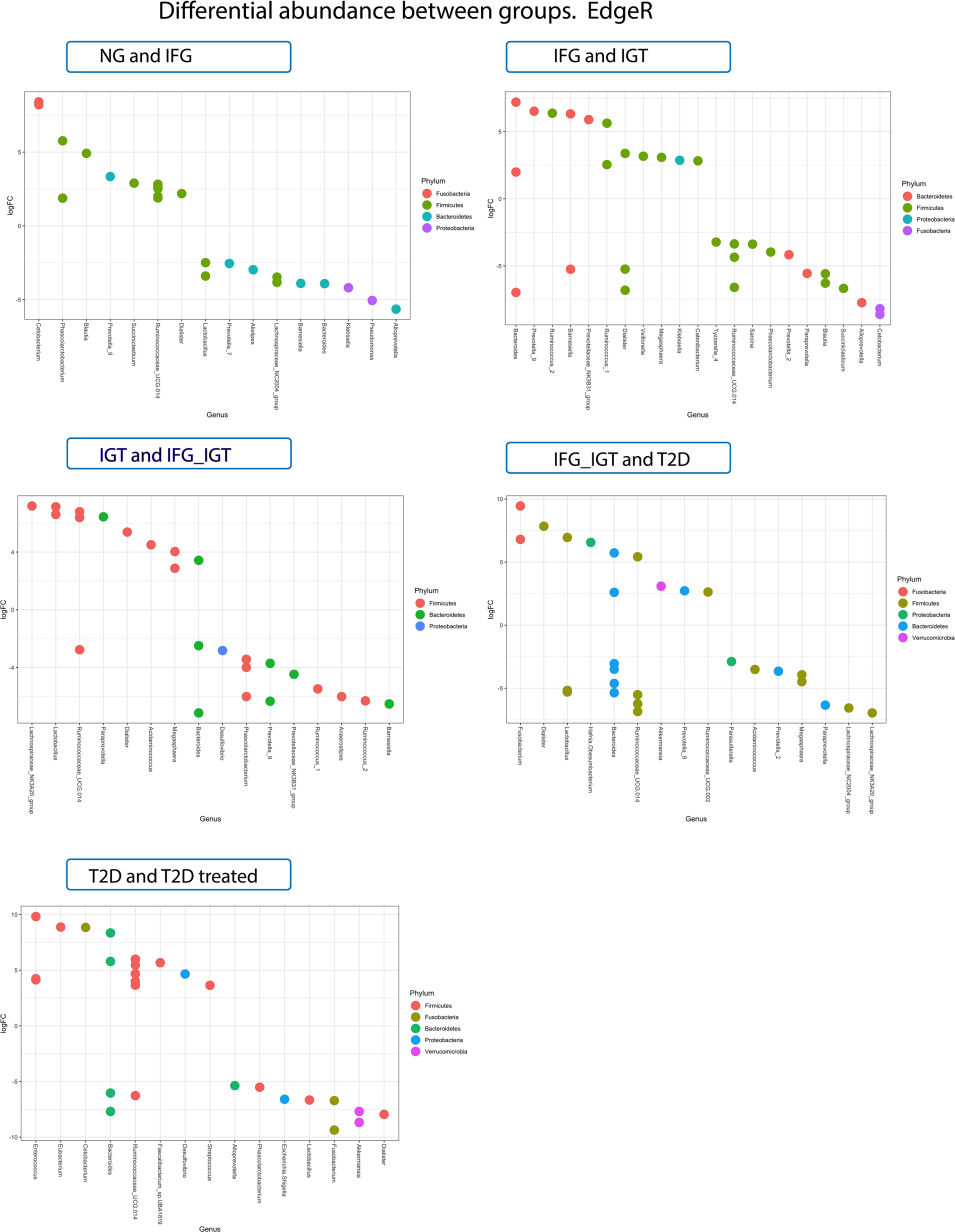
LogFC differential abundance (or coefficient from edgeR log-linear models for each comparison group and all significant ASVs) of GM data of Mexican T2D patients. Colors refer to the phyla taxonomy level of the plotted genera.

Numerous taxa were differentially abundant among T2D stages. **Figure 2** displays log-2 fold change (logFC). The logFC can be interpreted as the log-base-2 ratio of relative abundance compared to the reference group. For example, *Blautia* was found to be 32 (2^5^) times more abundant in IFG subjects compared with NG subjects, but in another comparison was found to be 36.75 (2^5.2^) times less abundant in IGT subjects compared with IFG subjects (**Figure 2**).

*Prevotella* 9 genus is another example of notable variations along preT2D stages. It was 36.75 (2^2.5^) more abundant in IFG patients compared with NG patients. Similar behavior was observed in other stages of preT2D, where it was 39.39 (2^5.3^) more abundant in IGT patients compared with IFG and 5.27 (2^2.4^) more abundant in T2D patients compared with IFG+IGT patients. However, it was 16 (2^4^) less abundant in IFG+IGT subjects compared with IGT patients (**Figure 2**). Notably, this is the first report where a genus-level cluster of *Prevotella* 9 emerges in a T2D cohort.

### 3.3 Association networks analysis to describe the GM insights among T2D stages

Modeling interactions by association networks is an effective computational tool for analyzing the structure and stability of microbial communities. Also, possible keystone taxa (driver taxa) related to the dynamic equilibria of GM and its host can be inferred. We performed and analyzed association networks for each stage of T2D (i.e., NG, IFG, IGT, IFG+IGT, T2D, and T2D treated). Then, we used Netshift to identify the driver nodes in each case-control association as those accomplished in the differential abundance analysis (NG vs. IFG, IFG vs. IGT, etc.) (**Figure S4A**). In each comparison, we built and compared the network association for each state and obtained some topological parameters, such as the network density, cluster coefficient, and average path length. Together, they are called global graph properties because they provide insights into the overall organization of the network and enable the assessment of its modularity (35).

In a microbial community, density corresponds to the proportion of observed microbial associations (edges) out of all theoretically possible associations (all the nodes in the network). Therefore, a greater density value indicates higher crosstalk among the resident microbes represented in the network nodes (35). Moreover, network density shows how quickly perturbations may spread (51). Thus, the small network density (**Figure S4A**) indicated microbial communities composed of scarcely connected groups. This behavior was expected due to the scarce resilience of the system since a poorly connected network is less robust to changes than high-density networks (52).

Complementary, it has been suggested that communities with the modular organization of the type “small world” are more stable at facing perturbations. The modular arrangement allows different groups of nodes to perform different functions with some degree of independence (53). In this way, the clustering coefficient quantifies the tendency of the graph to be divided into subunits. In other words, a microbial network with a higher number of independent units of associated microbes is expected to have a higher clustering coefficient value (35). Our study showed slight changes in this parameter between comparisons, particularly IGT vs. IFG+IGT and IFG+IGT vs. T2D had remarkable changes (almost double the previous value) (**Figure S4A**).

Average path-length indicates the average number of steps that would be required to reach from one node to another in the network. This parameter represents to what extent the microbial community structure is compacted. In almost all cases, we detected an increase in the path length from 1.5 to 2 in IGT vs. IFG+IGT, from 1.2 to 1.5 in NG vs. IFG, and IFG+IGT vs. T2D, for example (**Figure S4A**). However, we detected a large change in the T2D vs. T2D treated. Based on the previous results, low density and lower average path length in the network indicated lower information transport which might suggest a decolonization activity in the GM.

**Figure S4B** showed that the number of total nodes decreased on every pairwise comparison (i.e., IFG vs. IGT, IGT vs. IFG_IGT, IFG_IGT vs. T2D, T2D vs. T2D treated), except in NG vs. IFG where only remains the same number of total nodes (**Figure S4B**). These results agree with our previous results, where we suggested a possible decolonization activity in the GM along with T2D development and progression.

Regarding the total number of edges, we detected several exciting patterns (**Figure S4B**). For example, we observed that the number of total edges decreased for NG vs. IFG, IFG vs. IGT, and IGT vs. IFG_IGT, while for IFG_IGT vs. T2D and T2D vs. T2D treated increased. As expected, the comparison of T2D vs. T2D treated had a remarkable difference as the GM composition changed within T2D subjects treated with antidiabetic drugs such as metformin (54). In terms of exclusive edges, we detected patterns such as NG vs. IFG, IFG_IGT vs. T2D, and T2D vs. T2D treated with an increase in this value. From the other side, we detected a decrease in terms of exclusive edges for IFG vs. IGT and IGT vs. IFG_IGT. This behavior is in agreement with our results of density and average path length (**Figure S4A**). Moreover, it was previously observed in some pathologies related to GM, such as IBD (55).

Although the exclusive edge count between two networks is an indicator of rewiring, it is also valuable to consider the Jaccard Edge Index (JEI) of the compared networks as it can quantify changes in the interacting partners for each node between two graphs (each T2D stage) (**Figure S4C**). According to **Figure S4C** and in the first stage of the disease, we observed in the pairwise comparisons, NG vs. IFG and IFG vs. IGT, the value of JEI is near to 1 and indicated that the edges in the association networks are practically the same. But when the disease evolved to other stages such as IGT, IFG_IGT, T2D, and T2D treated, JEI decreased dramatically to zero. These results can be interpreted as a remarkable change owing to a different stage of T2D disease (56). In short, all these topological properties of the association networks offer insights into the overall differences in the community structure between a pairwise comparison network.

### 3.4 Driver nodes detection through Netshift and keystone taxa among T2D stages

**Figure 3:**
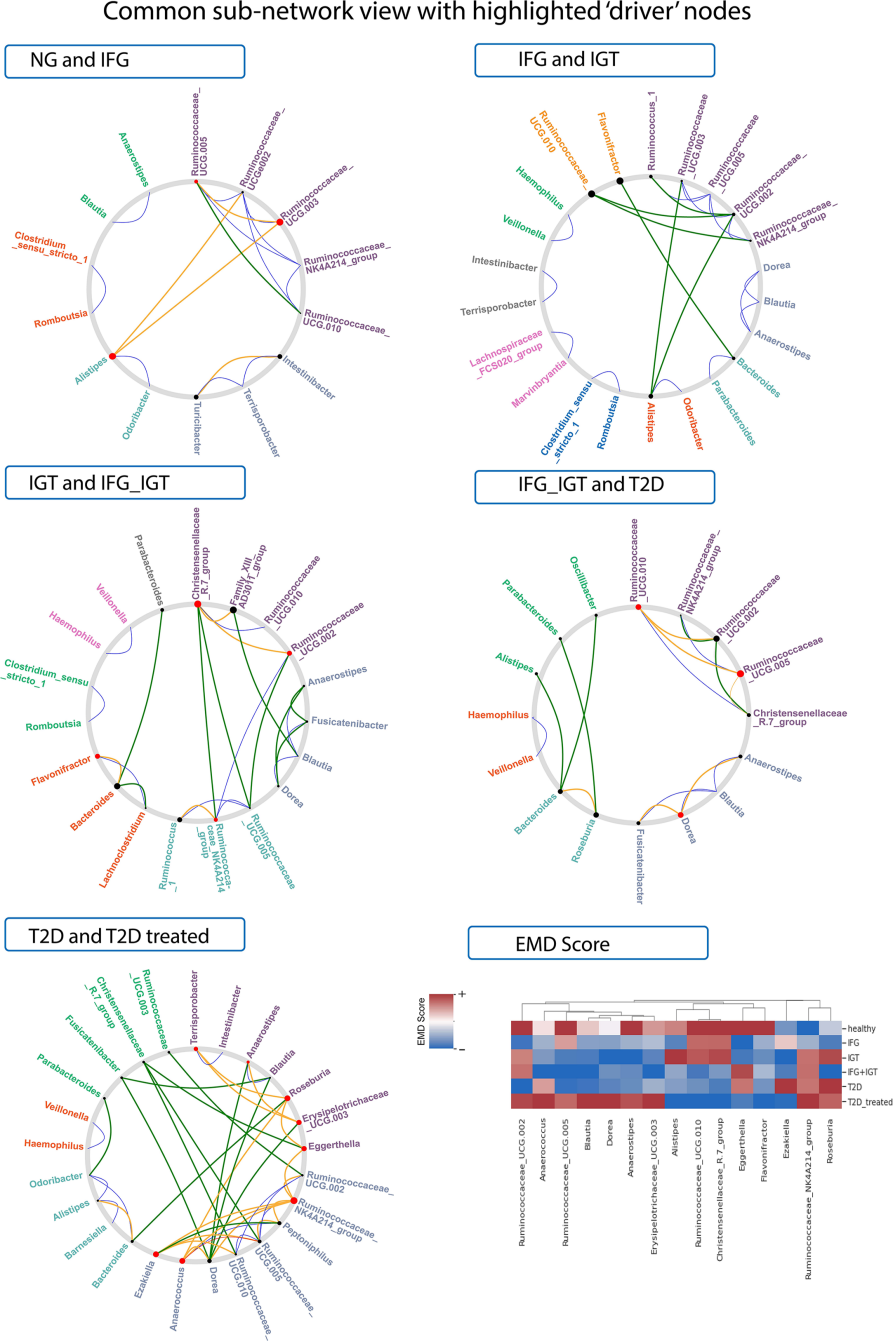
Driver nodes were obtained with NetShift. Driver nodes were colored red, edges present in both states were colored with blue, edges colored with green were exclusive to control data, and edges colored with red were exclusive to case data. All comparisons are based on a control-case order. Heatmap of the key drivers shifts among T2D states based on the denoised data, the earth-mover distance (EMD) is used to quantify distribution shifts among the T2D states clusters.

**Figure 4:**
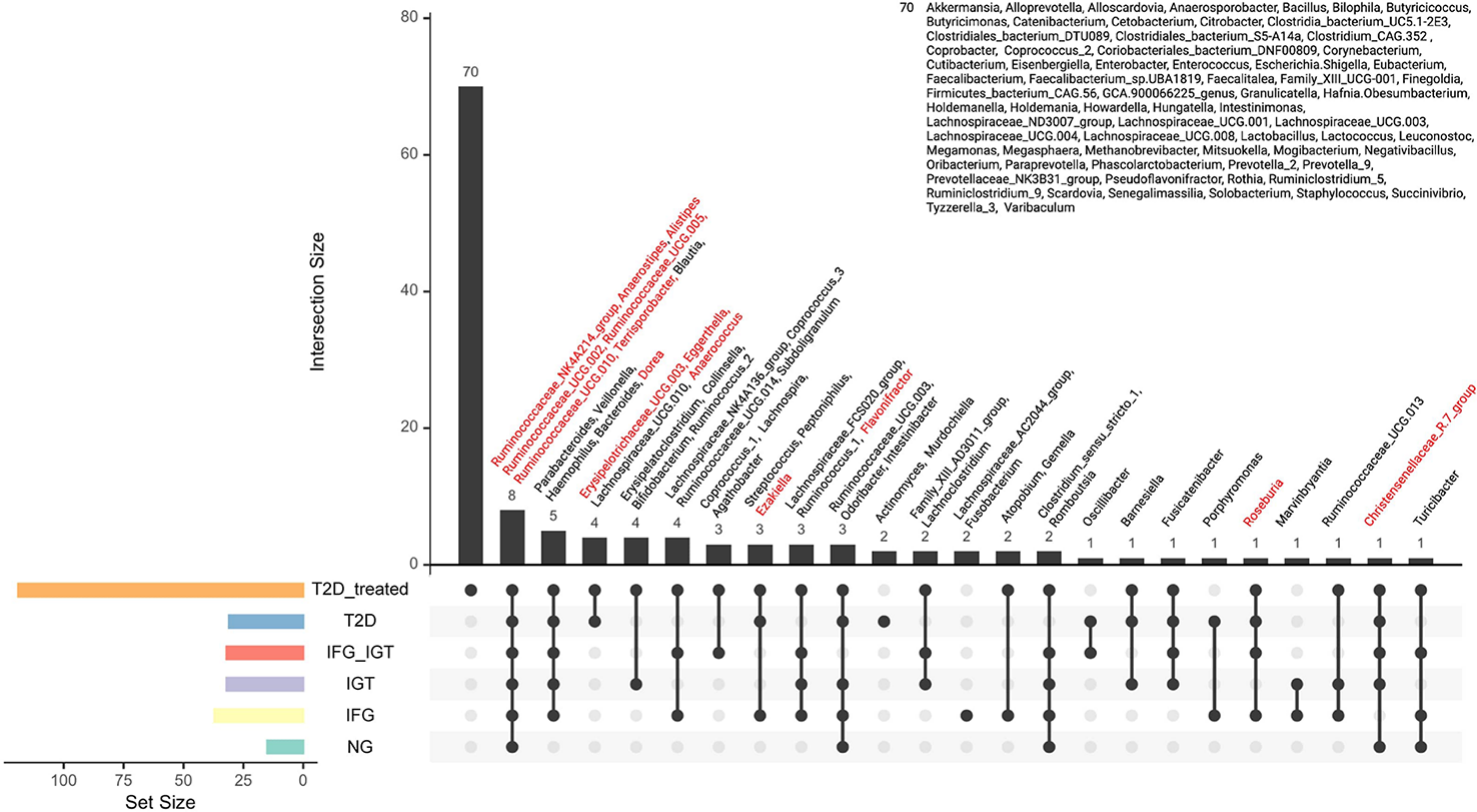
Core nodes for the Mexican T2D cohort. Each node represents a node in the SparCC association networks. The red color indicates “driver nodes” obtained from NetShift Analysis. Below, the black dots represent the presence of the core in the corresponding study group (e.g. T2D treated has 70 unique genera along with the cohort).

In many diseases, a set of key microbial groups are likely to act as ‘drivers’ for facilitating several changes in the microbial community structure and hence become an essential factor for understanding the microbial basis of the disease (35, 55–59). With this idea in mind, we obtained the driver nodes for each pairwise comparison with the software Netshift (**Figure 3**). **Figure 3** shows our results of driver nodes for the pairwise comparisons analyzed along with this study. These keystone nodes belong to several phyla: 1) *Anaerococcus, Anaerostipes, Christensenellaceae, Dorea, Ezakiella, Erysipelotrichaceae, Roseburia, Terrisporobacter*, and *Ruminococcaceae* belonging to *Firmicutes* phylum. 2) *Alistipes* belonging to *Bacteroidetes* phylum, and 3) *Eggerthella* belonging to *Actinobacteria* phylum. Our results fall into the most representative genera and phyla belonging to GM (49) and add more evidence to our previous hypothesis, which proposes that the changes in the human gut enterotypes are associated with some keystone or driver taxa along the different stages of preT2D and T2D in a Mexican cohort. Later, we prepared an Upset Plot graph with all the nodes obtained with SparCC and the driver nodes from NetShift to determine specific genera that belong to a particular group (**Figure 4**). As seen in **Figure 4**, *Ruminococcaceae_*NK4A214_group*, Anaerostipes, Alistipes, Ruminococcaceae_*UCG-002*, Ruminococcaceae_*UCG-005, *Ruminococcaceae_*UCG-010, and *Terrisporobacter* were the core community in all analyzed groups. This finding is highly relevant as it is the first microbial core reported for the GM of Mexicans with T2D.

### 3.5 Multidimensional approach by bioinformatics, microbial ecology, and denoising microbiome tools to identify keystone taxa and their interactions among T2D stages

**Figure 5:**
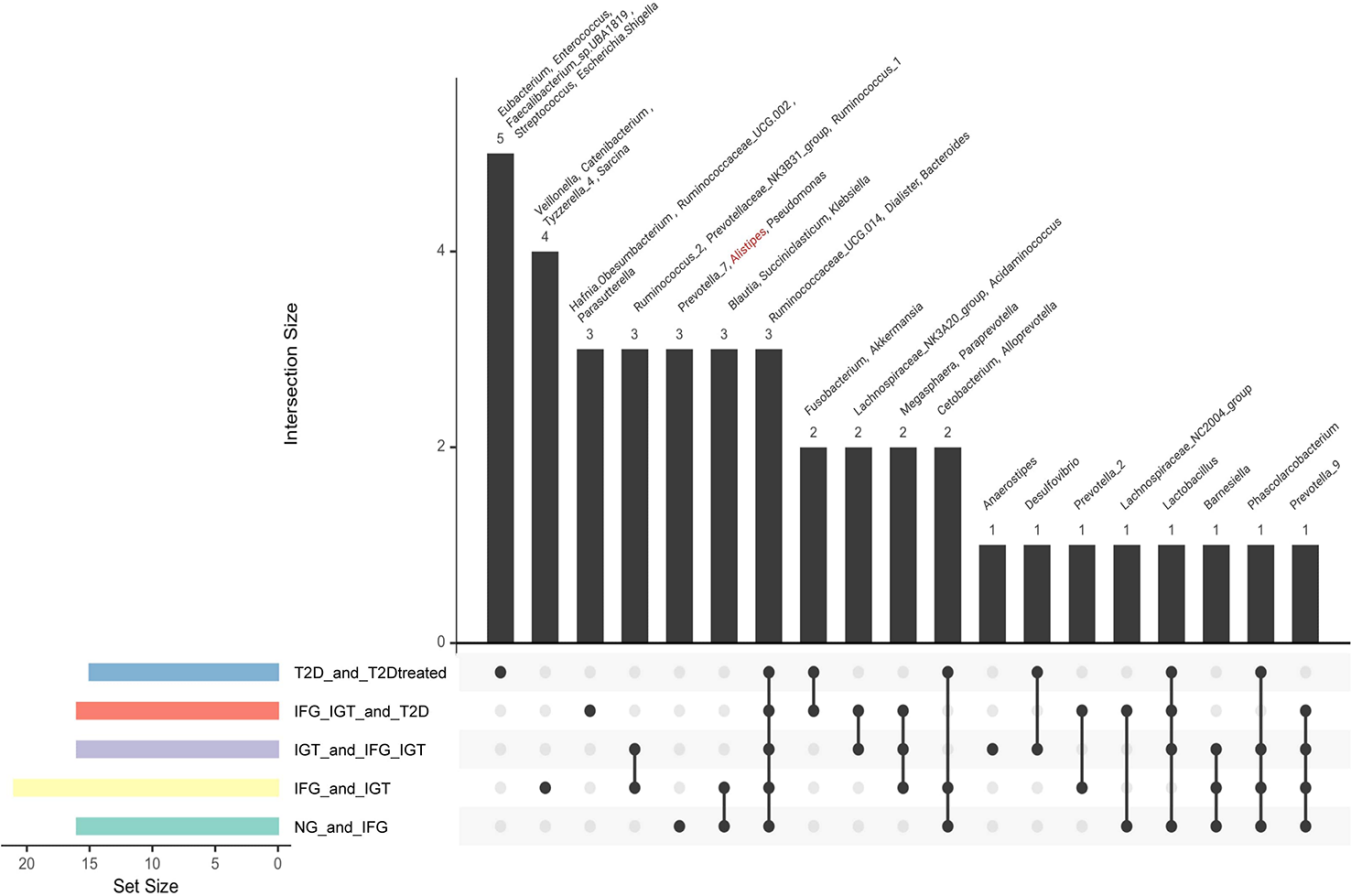
A multidimensional approach to interlace nodes information obtained with the differentially abundance tool (EdgeR) and driver nodes (Netshift). For both EdgeR and Netshift, the data was obtained from pairwise comparisons along with the cohort. NG vs IFG, IFG vs IGT, IGT vs IFG_IGT, IFG_IGT vs T2D and T2D vs T2D treated. *Note: In red were marked genera that had “driver node” characteristic from NetShift Analysis.

To analyze in-depth all these data from networks, we compared the results of differentially abundant analysis of all groups in the cohort (**Figure 2**) with the results from NetShift (**Figure 3**) to get coincidences between these two approaches (**Figure 5**). *Alistipes* can be a potential SCFA producer, and their decrease contributes to the development of inflammatory diseases related to inadequate modulation of the immune system by SCFA in the gut (e.g., CRC, IBD, cardiovascular disease (CVD), etc.) (60, 61). We detected a remarkable effect of *Alistipes* through two different approaches: 1) Association networks with SparCC and Netshift analysis and 2) Differential Abundance for Microbiome Data with EdgeR. Based on these results, *Alistipes* seems to play a role in the development of preT2D (IFG) as with other inflammatory diseases such as IBD, CRC, CVD, and hypertension (62).

**Figure 6:**
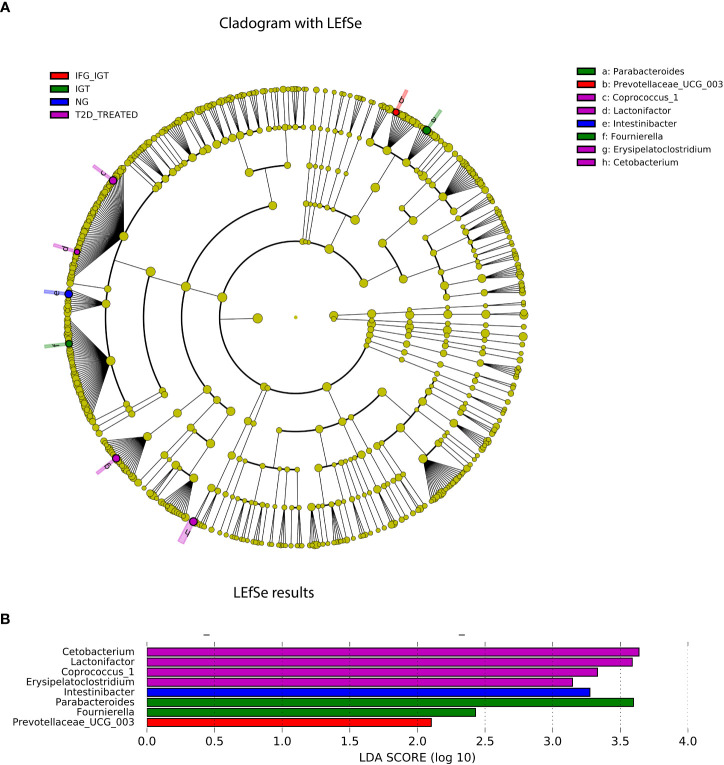
LEfSe analysis results. **(A)** Taxonomic cladogram. Differences are represented by the color of the most abundant genera at each group (NG, IFG, IGT, IFG+IGT, T2D and T2D treated). The diameter of each circle is proportional to the taxon's abundance. **(B)** The linear discriminant analysis (LDA) value histogram of the GM at genus level and cohort groups (NG, IFG, IGT, IFG+IGT, T2D and T2D treated).

To reinforce the results obtained with Netshift about the taxa-drivers in each T2D stage (**Figures 5**, **6**), we denoised microbiota data (ASV table) through mb-PHENIX to remark the potential driver nodes involved in the transition of T2D states (37). Specifically, we used this approach because data lacks a well-defined cluster structure (**Figures S3A, B** (NMDS), **Figure S5A**, (UMAP)) due to the high rate of missing information (38). Then, we quantified the differences of the recovered (denoised) taxa between different T2D stages with EMD (63). We observed statistically relevant changes in the abundance of a determined driver or keystone taxa (**Figure 3** EMD Score) and confirmed our previous results with EdgeR, SparCC, and Netshift. Hence, we used several tools to prove the existence of keystone taxa pattern shifts among T2D stages. **Figure S5B** shows that the changes along the T2D stages were mediated by a group of microbial interactions independently of whether their abundance is significantly different or not in a specific T2D group (**Figure S5C**). Therefore, this work showed the complexity of intrinsic non-linearity and the potential taxa differentiating the different groups of T2D states.

### 3.6 Key bacterial genera changes among T2D stages in a Mexican cohort

To further explore differences in the GM among our groups (NG, IFG, IGT, IFG+IGT, T2D, and T2D treated), we used LEfSe to recognize the specific altered bacterial phenotype at genus level. These differences are shown in **Figure 6**. For the T2D treated group, we detected four bacterial genera that changed significantly among the groups with LDA score log 10 > 2, *Cetobacterium, Lactonifactor, Coprococcus_1*, and *Erysipelatoclostridium*. For the IGT group, we detected two bacterial genera with the same LDA score, *Parabacteroides* and *Fournierella*, while for the NG and the IFG+IGT groups, we detected *Intestinibacter and Prevotellaceae_UCG003*, respectively.

## 4 Discussion

T2D is an expanding global health problem closely linked to obesity, and hypertension (9). Changes in the structure and composition of GM in T2D patients represent a key step to understanding the GM dysbiosis that accompanies the progression of IR in T2D. In this way, the GM is a complex system that needs to be analyzed in a holistic way, with several approaches such as bioinformatics and systems biology altogether under the concept of medical ecology of the human gut microbiota.

In terms of ecological indexes (α and β), we detected some variations related to the weight of the patients and their group (i.e. NG, IFG, IGT, IFG+IGT, T2D, and T2D_treated). This phenomenon is probably connected with the role of GM in T2D and its related disorders, including obesity. In agreement with this observation, some studies in murine obesity models indicated that altered GM had an increased capacity to harvest energy from the diet (64, 65) and suggested that changes in the diversity of GM should be considered a contributing factor to the pathophysiology of obesity and T2D.

Furthermore, the keystone roles of *Prevotella* 9 and *Blautia* with T2D progression were remarkable. In the specific case of *Blautia*, several studies have reported similar roles in other human diseases: fewer *Blautia* in T2D/obesity patients, fewer in colorectal cancer (CRC) and Inflammatory bowel disease (IBD) (66). This is relevant because, as far as we know, this is the first report of differential abundance changes of *Blautia* in IFG and IGT. Specifically, *Prevotella (Bacteroidetes)* and *Blautia (Firmicutes)* are remarkable examples in this analysis, which both suggest a subject-specific microbiome type led to the concept of human gut enterotypes that may change among NG and T2D groups of our Mexican cohort (67, 68). Overall, the study of *Prevotella* and *Blautia* has been hampered by their intrinsic difficulty in cultivating *In vitro* (they are anaerobic) (66, 69).

Network analysis is a powerful tool to understand the structure and ecological functions of the GM in patients with T2D. In the Netshift analysis, we detected a core of genera present in our Mexican T2D cohort. This core captures several ecological insights that will be explained next. *Ruminococcaceae_*NK4A214_group genus has been related to fiber degradation (58). In a murine model, this genus was correlated with alleviating colitis in casein-fed mice (70). However, another study with rats showed this genus with positive correlations with levels of blood glucose, HOMA-IR, and lipopolysaccharides (LPS) (71). Also, other studies showed increased abundance in a cohort of obese adults with elevated fasting glucose levels subjected to an almond consumption diet; interestingly, this genus was identified as one of the principal drivers of microbiota-level changes in our cohort (56).

In agreement with our findings, *Alistipes* (SCFA producer) was also increased in a T2D Austrian Cohort (72). Several authors reported that high levels of *Alistipes* and low levels of *Blautia* were found in patients with T2D (73). Moreover, *Alistipes* abundance was increased in diabetic mice with a potential association with high sucrose and a high-fat diet (74, 75). Whereas, in hypertension, it seems that *Alistipes* contributes to inflammation and epithelium alterations as *A. finegoldii* had an increased number and functional genes in the high blood pressure cohort (62). These results indicate that the composition of the GM is closely related to the levels of blood glucose and pro-inflammatory cytokines, which cause low-grade inflammation and contribute to the T2D progression. In this scenery, IR could be a consequence of dysregulation of bacterial production of butyrate, SCFA, and other metabolites (73).

The genus *Anaerostipes* (SCFA producer) was found significantly decreased in T2D cases compared to controls in an African cohort, further, the GM of T2D patients had decreased butyrate-producing bacteria and consequently reduced butyrate production, previously associated with IR (76). Several studies showed that *Anaerostipes* could interact with other microbes with diverse catabolic capacities to produce lactate (77). To this extent, some species of *Anaerostipes* (e.g., *A. caccae, A. rhamnosivorans, A. hadrus, and A. butyraticus)* are not only able to use a broad range of carbohydrates but also lactate and acetate for butyrogenesis (78). Nevertheless, to disentangle the metabolic differences between species and strains, we need to obtain broad genomic information through shotgun metagenomics.

Following up with the core microorganisms, a study in obesity and fasting plasma insulin (FPI) status in Mexican children reported a negative association of obesity with *Ruminococcaceae* UCG-002 and a positive association between FPI and *Ruminococcaceae* UCG-002 (79). For *Ruminococcaceae* UCG-005, some authors reported a positive correlation with *Christensenellaceae* R-7-group genus abundances and HDL cholesterol but a negative correlation with triglyceride levels (21). As mentioned before, the genera *Ruminococcaceae* UCG-010 showed a positive correlation with the levels of blood glucose, HOMA-IR, and LPS in diabetic (T2D) rats (71). Bacterial members of this family predominantly utilize fibers and polysaccharides as energy substrates and are SCFA producers. SCFAs are linked to improved colonic health and are known mainly for their anti-inflammatory properties (80). However, the relevance of *Ruminococcaceae* to health outcomes has not been fully elucidated. Conflicting data show that these genera are both positively and negatively correlated with lipid metabolites such as VLDL and HDL and are also associated with higher BMI (81).

*Terrisporobacter* was related to inflammation and oxidative stress in a study of NG Danish young men. This genus decreased immediately after metformin treatment initiation and remained low throughout the intervention period (82). In a similar report with Egyptian patients, authors reported that *Terrisporobacter* and *Turicibacter* were significantly more abundant in the control group (NG) as compared to either Type 1 or Type 2 Diabetes groups (83).

*Dorea* also appeared as a core for patients in all stages of the disease (i.e., IFG, IGT, IFG_IGT, T2D, and T2D treated). A Spanish cross-sectional study reported that body weight, waist circumference, and BMI showed a positive association with *Dorea formicigenerans* and *Dorea longicatena* with increased abundances in the overweight/obese group. They proposed these species as microbiota biomarkers of obesity in the Spanish population (84). These results agree with another study in a Chinese population, where the abundance of *Dorea* was significantly increased in T2D individuals and negatively correlated with the abundance of butyrate-producing bacteria. The authors argued that increases in *Dorea* could play a role in the development of T2D (85). Supporting this, a cohort study of Danish patients with preT2D (IFG) and NG individuals reported a differentially significant increase of Dorea in the IFG patients versus NG individuals (10).

*Roseburia* is known to be a butyrate-producing genus, dependent on fermentable carbohydrates from the diet (82). In our results, *Roseburia* appeared as a core and driver node of all stages of T2D, except for the IGT group. Thus, it is plausible that *Roseburia* could affect T2D development. Still, its specific role is unclear since in our study appeared in the groups with T2D or preT2D and in other studies appeared as a health biomarker (6, 86).

*Blautia* is a particular case because it was not identified as a driver genus in Netshift but appeared as a core genus in the cohort. This behavior can be explained by its increase in disease groups in three of four cross-sectional studies of T2D and its reduction after bariatric surgery (6). Disagreeing with these reports, *Blautia* spp. was increased after metformin treatment (17). Notably, 87 results are concordant with our genus-level analysis, demonstrating positive associations between T2D and several OTUs of all three of these genera (87, 88).

In accordance with the LEfSe analysis, all identified bacteria belong to genera with previous reports linked to the progression of insulin resistance in T2D (**Figure 6**). However, *Coprococcus_1*, found in the T2D treated group, was already characterized by a high insulin sensitivity (β = 0.14; P = 0.002) and disposition index (β = 0.12; P = 0.012); for this reason, we hypothesized that its effect was mostly related to the changes in the constant metformin consumption in T2D patients (89). Furthermore, this is the first association of *Fournierella* with T2D patients and metformin treatment. Since this genus has reports with other low-grade inflammatory processes (i.e., obesity, insulin resistance, and others) that are part of the metabolic syndrome diagnostic (90–92). Another potential biomarker for the NG group was *Intestinibacter genus*. Early reports showed that a decrease in this bacteria abundance was described in patients with T2D (13).

According to our multidimensional analysis, *Alistipes* can be a potential biomarker of the development of T2D. Still, further studies will be necessary in the near future to better define the role of this genus when T2D is accompanied by other comorbidities (93). For instance, it has been reported that T2D patients have higher levels of depression incidence (94). There are several studies on mental diseases that reported an increase of *Alistipes* abundance (almost 4-fold) in Norwegian patients with chronic fatigue syndrome (95). These findings correspond with the evidence of an increase in T2D patients with depression, who typically struggle with fatigue and stress (96). An increase in *Alistipes* disrupts the gut-brain axis because it is an indole-positive organism and thus decreases serotonin availability. Tryptophan (indole ring) is a precursor of serotonin, and a decrease in serotonin is associated with depression. Moreover, *Alistipes* express glutamate decarboxylase in chickens, an enzyme that metabolizes glutamate into γ-aminobutyric acid (GABA). Therefore, an increase in *Alistipes* abundance can be related to a GABA increase with a potential link to depression (62). To confirm the role that *Alistipes* and other microorganisms have in comorbidities associated with T2D, additional studies should be addressed shortly.

## 5 Conclusion

We conclude that using bioinformatics, systems biology, and supervised machine learning tools, we obtained a complete ecological perspective of GM dysbiosis in a Mexican T2D cohort. *Ruminococcaceae NK4A214 group, Ruminococcaceae UCG-010, Ruminococcaceae UCG-002, Ruminococcaceae UCG-005, Alistipes, Anaerostipes*, and *Terrisporobacter* appear to have a distinctive ecological role in the preT2D group. However, more high throughput sequencing methods are needed to determine which species and strains are involved. Our arguments and results are important advances in the medical ecology of the human gut microbiota from a Mexican T2D cohort, as an effort to provide cutting-edge interventions for personalized medicine in the near future.

## Data availability statement

Raw sequencing data along with metadata is provided on the sequence read archive (SRA) under the Bioproject PRJNA541332 at https://www.ncbi.nlm.nih.gov/. The datasets generated and analyzed during the current study, and the customized scripts used in this study are available in the GitHub repositories: [https://github.com/resendislab/MEXT2D_Networks], [https://github.com/resendislab/mb-PHENIX],[https://github.com/resendislab/mext2d].

## Ethics statement

The studies involving human participants were reviewed and approved by Comité de Etica, Universidad de Guanajuato. The patients/participants provided their written informed consent to participate in this study.

## Author contributions

DE-H and OR-A conceived and designed the study. DE-H executed the experiments, analyzed the data, performed statistical tests, and drafted/revised the manuscript. YL-M, JS-C, DN-R, and CP-M analyzed the data, performed statistical tests, and contributed to the design of the research. DG-V and CM-O performed statistical tests, executed complementation experiments, and contributed to the implementation of bioinformatics analysis. All authors contributed to the writing of the manuscript and approved the final version.
