# Supplementary material for: A network perspective on the ecology of gut microbiota and progression of type 2 diabetes: Linkages to keystone taxa in a Mexican cohort
Source: Front Endocrinol (Lausanne). 2023 Apr 12;14:1128767. doi: 10.3389/fendo.2023.1128767 (PMC10130651; doi:10.3389/fendo.2023.1128767)
Supplement: Supplementary file 1 [file DataSheet_1.pdf]

Supplementary Material

1 Supplementary Figures

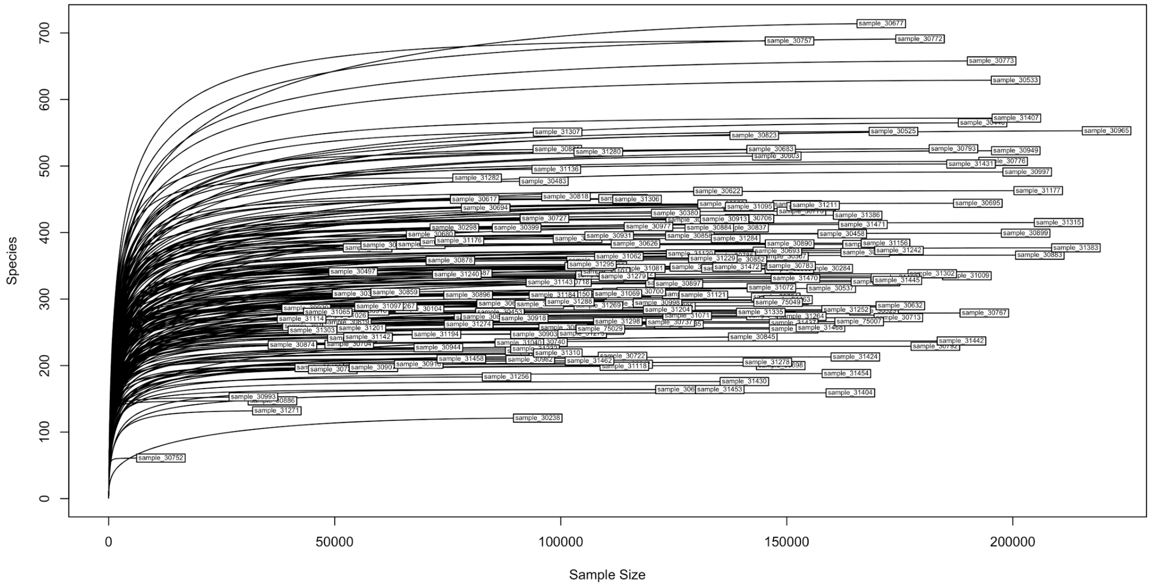

Figure S1-A. Rarefaction curve for healthy patients

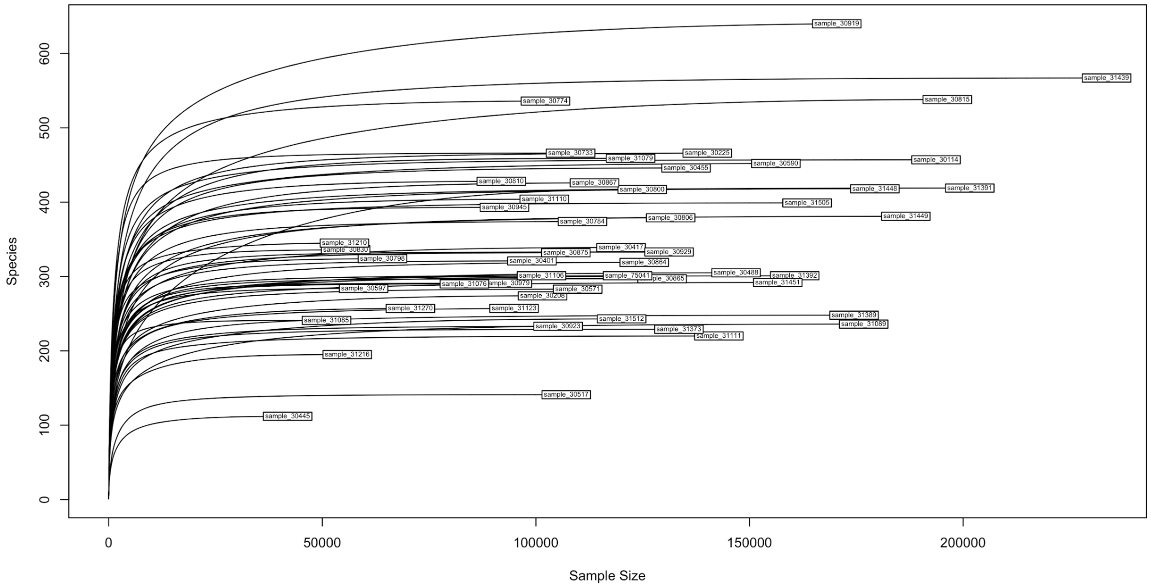

Figure S1-B. Rarefaction curve for IFG patients

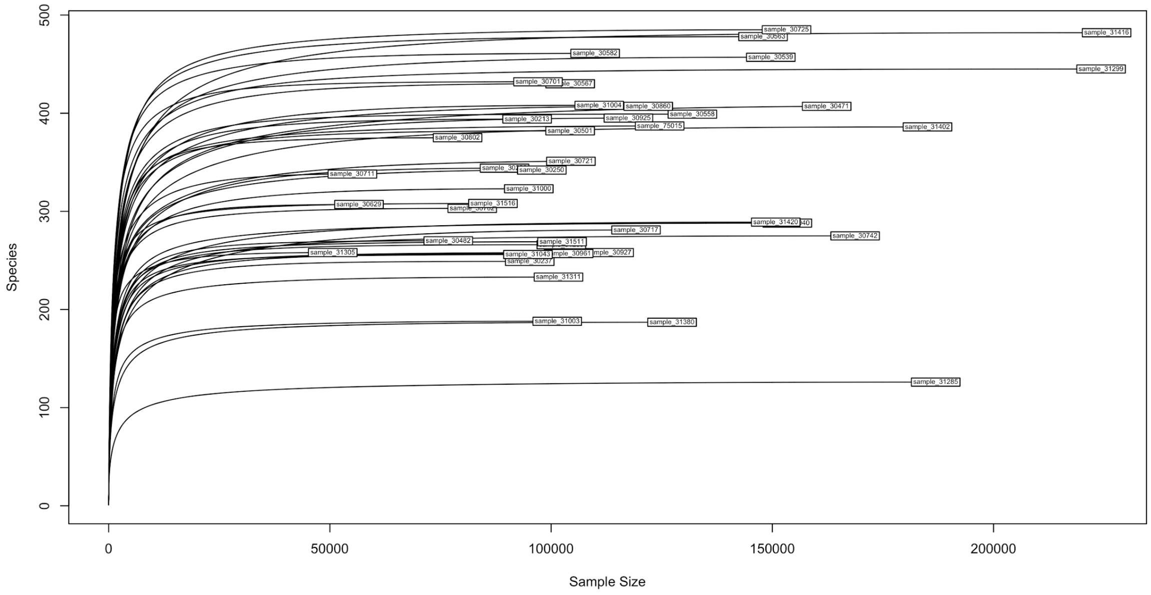

Figure S1-C. Rarefaction curve for IGT patients

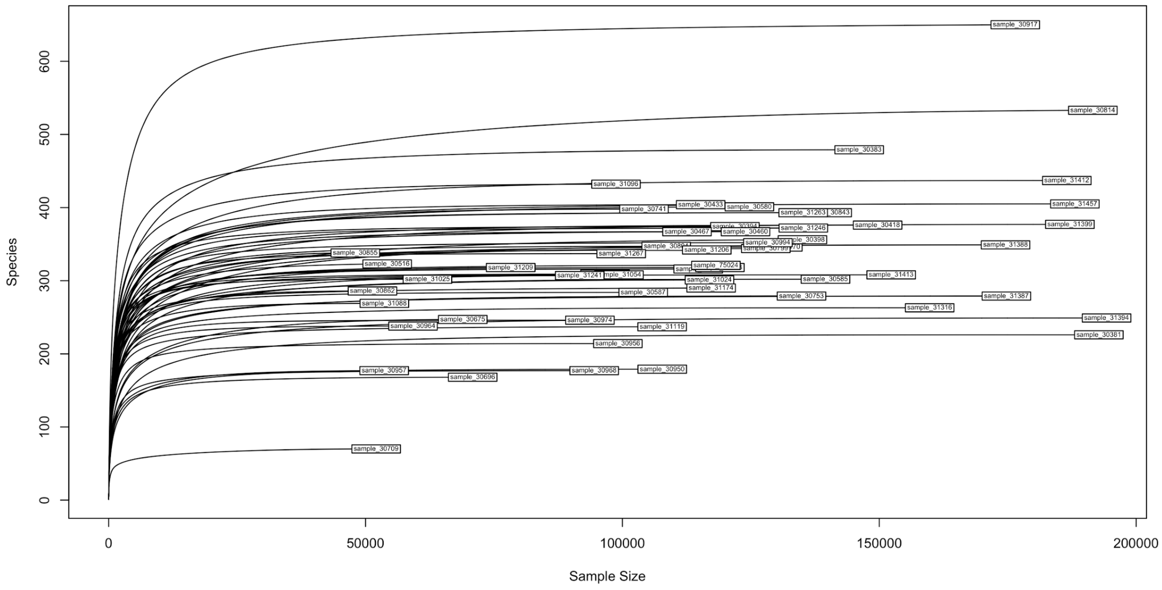

Figure S1-D. Rarefaction curve for IFG+IGT patients

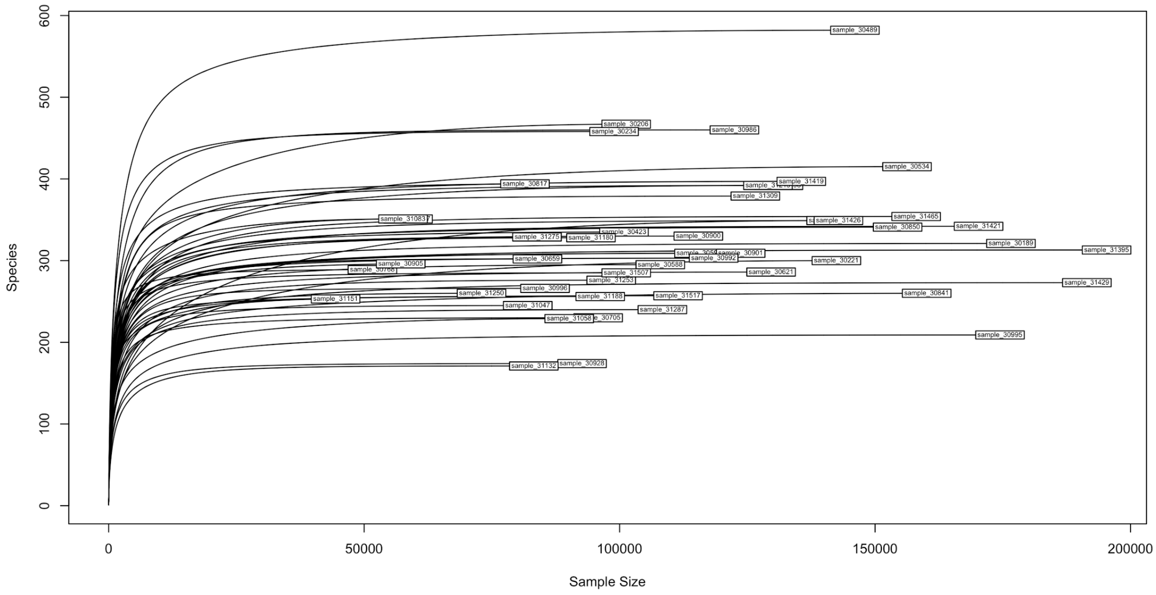

Figure S1-E. Rarefaction curve for T2D patients

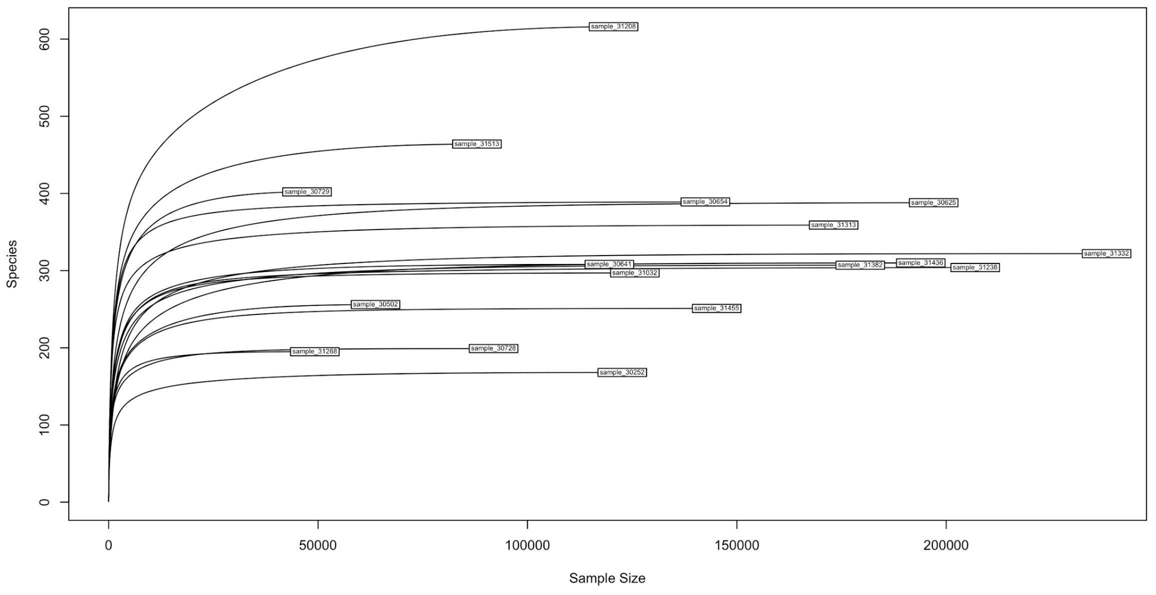

Figure S1-F. Rarefaction curve for T2D_treated patients

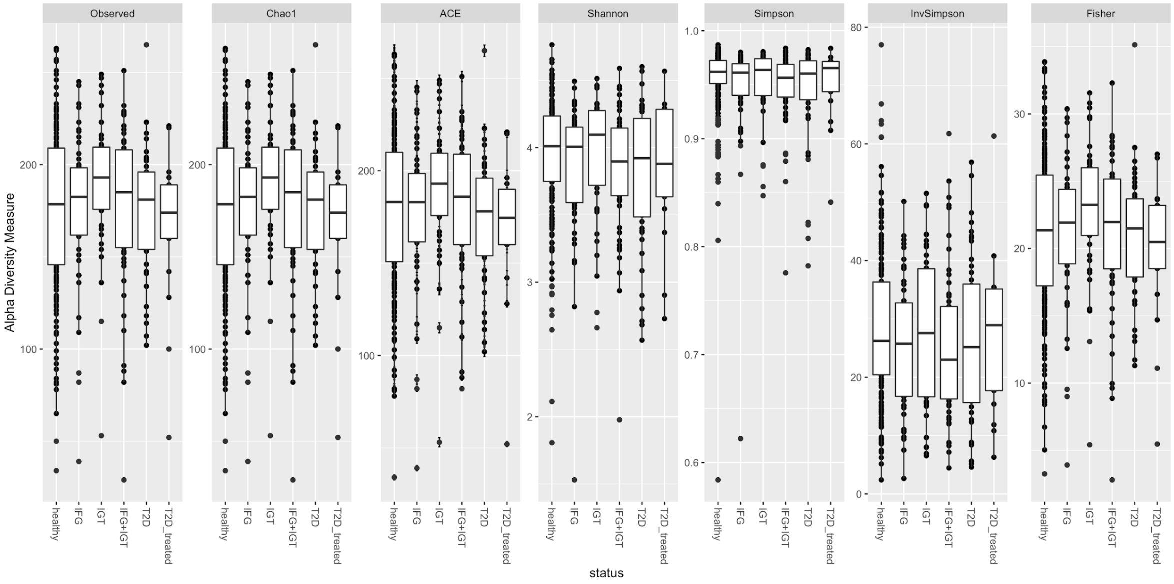

Figure S2. Alpha diversity index for all groups: Healthy, IFG, IGT, IFG+IGT, T2D, and T2D_treated

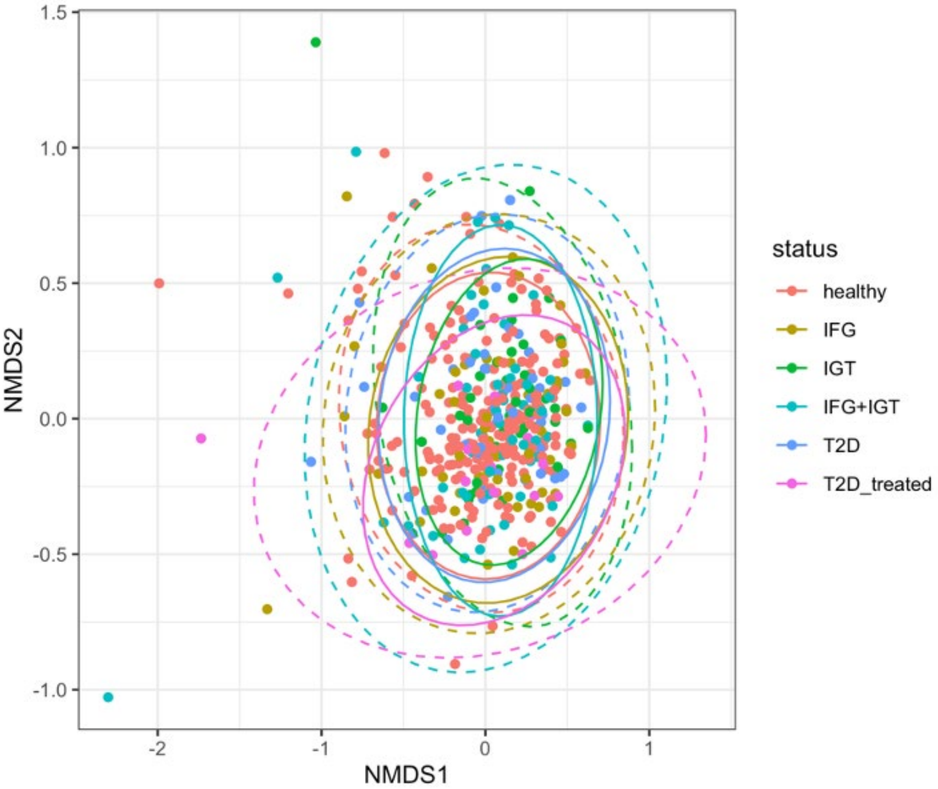

Figure S3-A NMDS analysis colored by groups: Healthy, IFG, IGT, IFG+IGT, T2D, and T2D_treated.

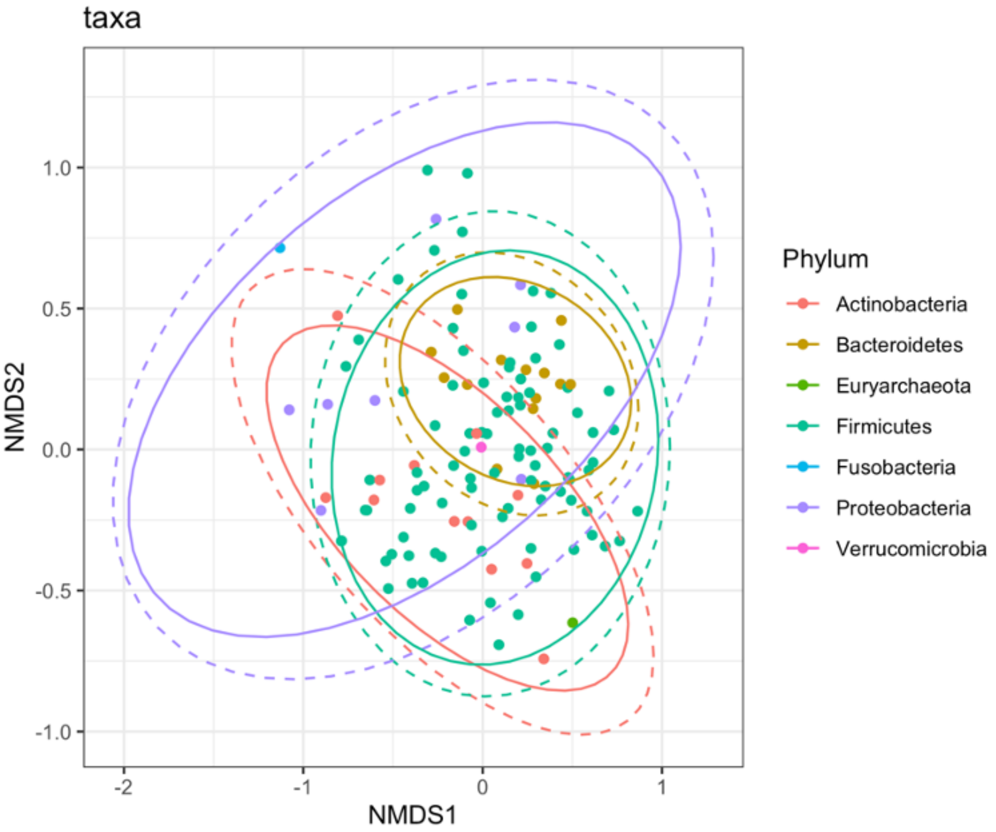

Figure S3-B NMDS analysis colored by phyla: *Actinobacteria, Bacteroidetes, Euryarchaeota, Firmicutes, Fusobacteria, Proteobacteria, Verrucomicrobia*.

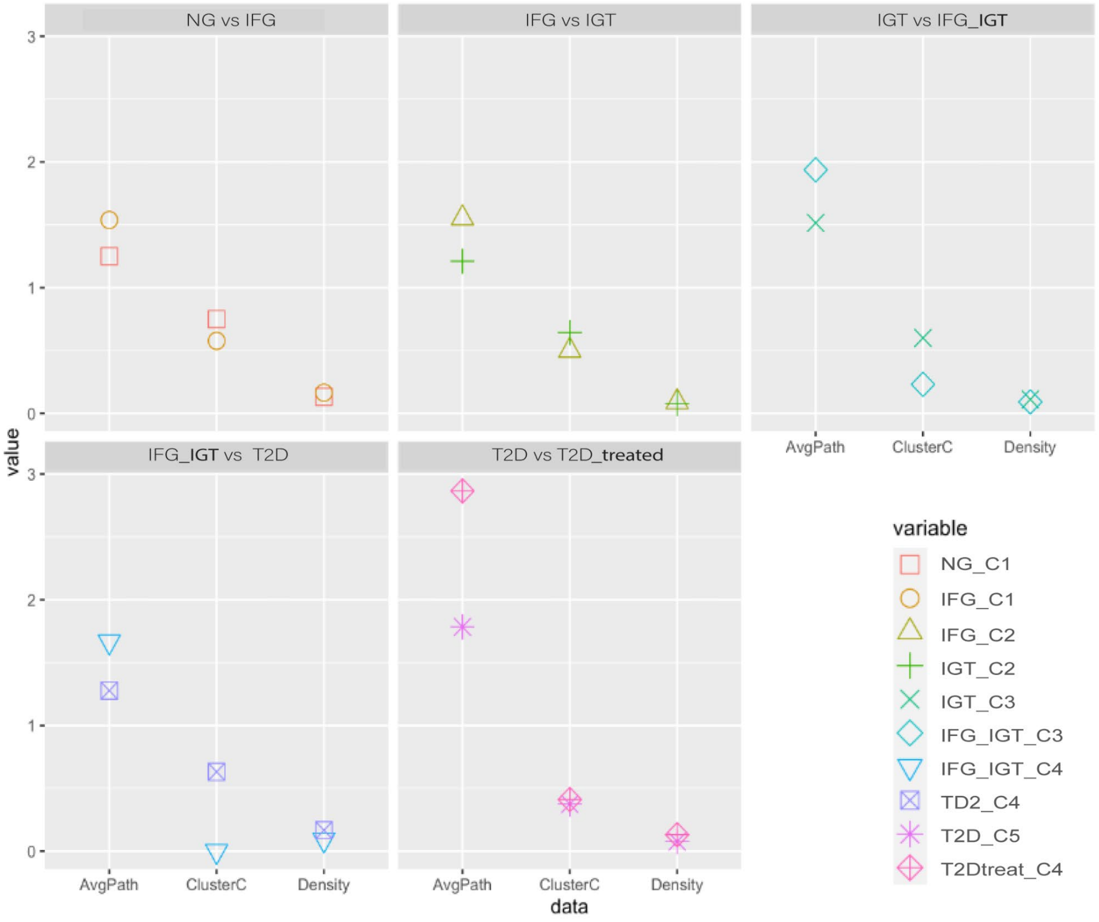

Figure S4-A Network statistics for all pairwise comparisons between groups (NG, IFG, IGT, IFG+IGT, T2D, and T2D treated). The X-axis indicates network statistics (AvgPath: Average Path, ClusterC: Cluster coefficient, and Density) while Y-axis indicates their corresponding values.

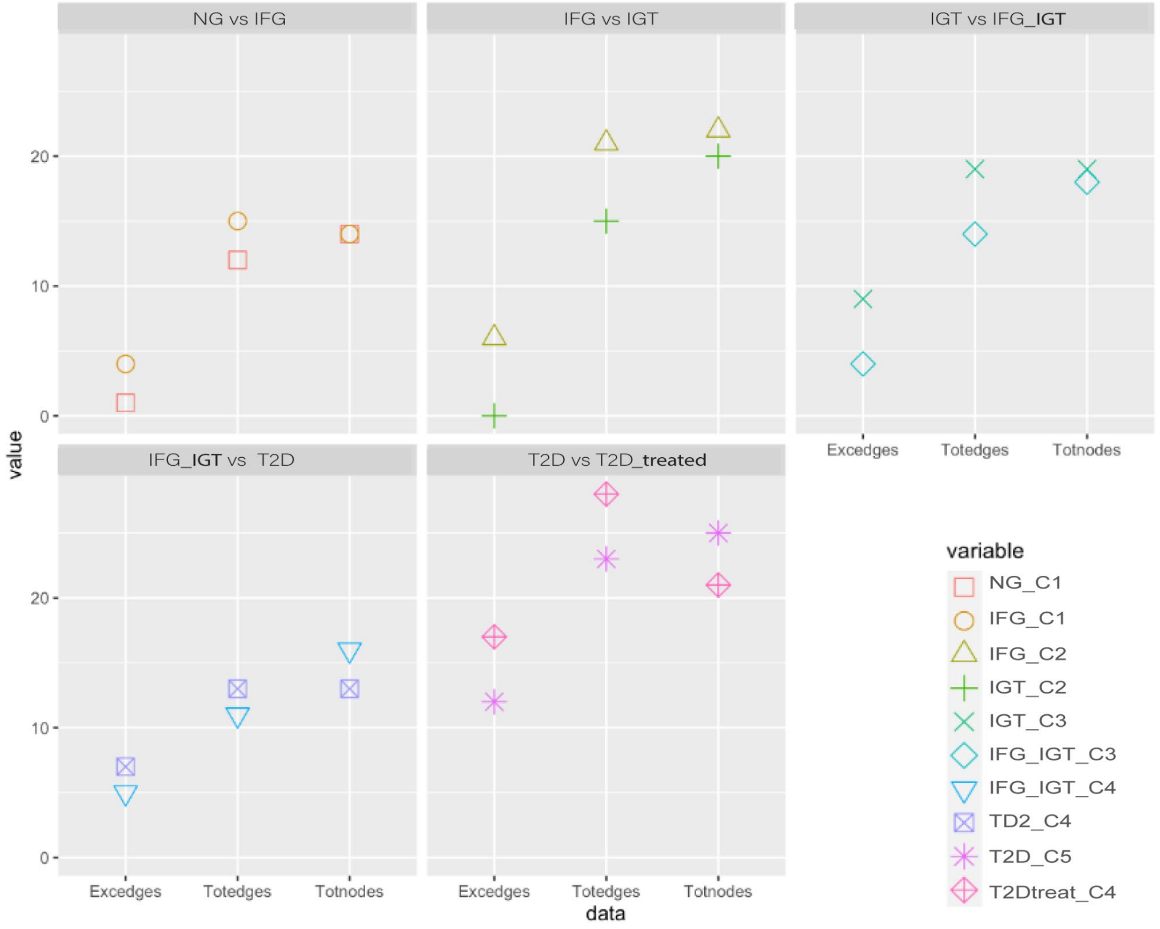

Figure S4-B Network statistics for all pairwise comparisons between groups (NG, IFG, IGT, IFG+IGT, T2D, and T2D treated). The X-axis indicates network statistics (Excedges: Exclusive edges, Totedges: Total edges, Totnodes: Total nodes) all of these are for each network while Y-axis indicates their corresponding values.

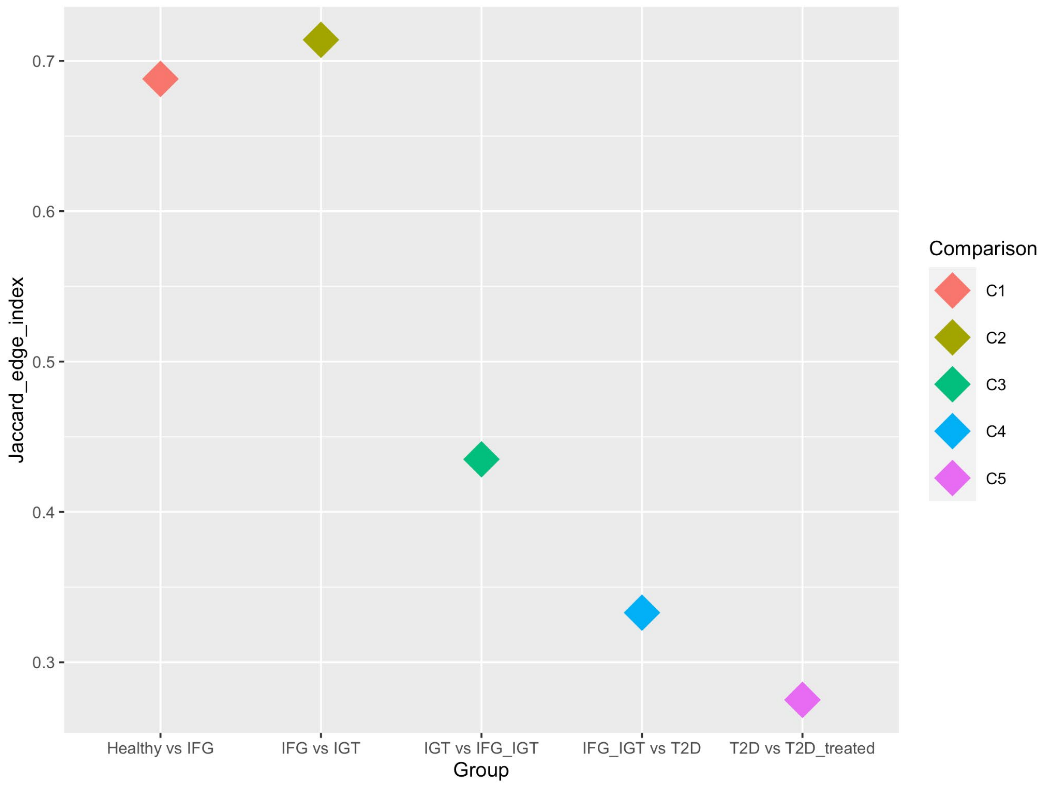

Figure S4-C. Jaccard Edge Index (JEI) for all pairwise comparisons in the T2D Mexican cohort.

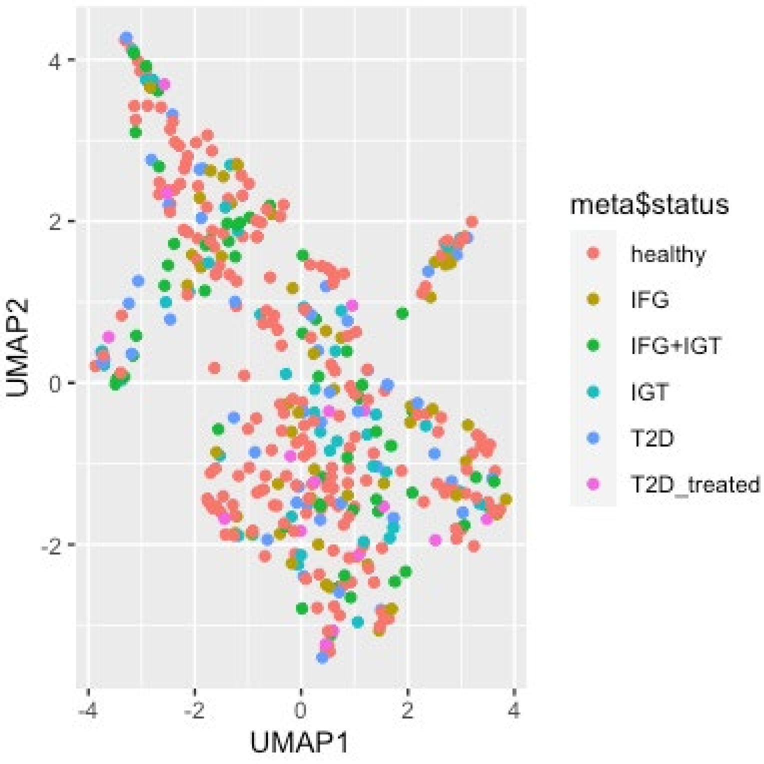

Figure S5-A UMAP analysis colored by groups: Healthy, IFG, IGT, IFG+IGT, T2D, and T2D_treated.

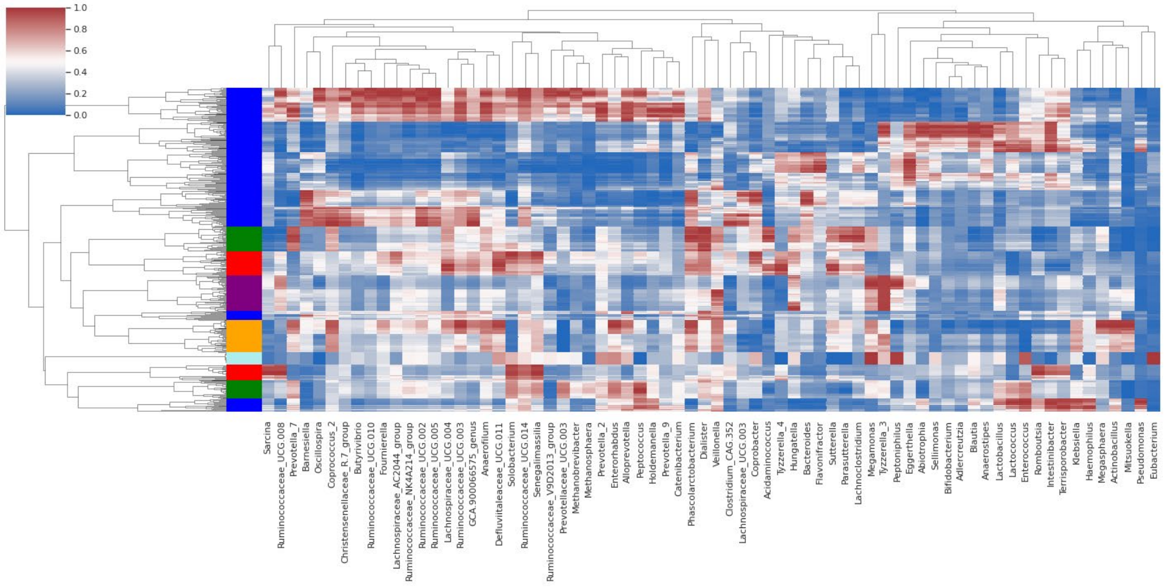

Figure S5-B Hierarchical analysis of the data imputed by mb-PHENIX imputed data.

We observed the top variable taxa (`percentile=60`) relevant abundance taxa data.

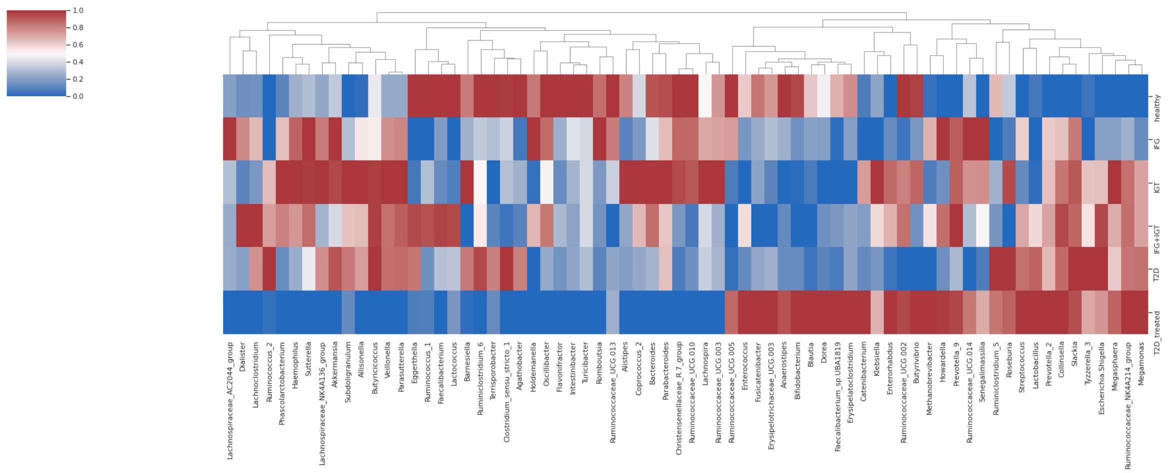

Figure S5-C Hierarchical analysis of the top 70 most relevant taxa before mb-PHENIX imputation among T2D states.

.
